# The regulatory role of N6-methyladenosine RNA modification in gastric cancer: Molecular mechanisms and potential therapeutic targets

**DOI:** 10.3389/fonc.2022.1074307

**Published:** 2022-12-06

**Authors:** Gaofeng Li, Qiru Fu, Cong Liu, Yuxi Peng, Jun Gong, Shilan Li, Yan Huang, Haiyuan Zhang

**Affiliations:** ^1^ School of Basic Medicine, Health Science Center, Yangtze University, Jingzhou, Hubei, China; ^2^ Editorial Department of Journal of Hubei University of Science and Technology, Xianning, Hubei, China; ^3^ Department of Abdominal and Pelvic Medical Oncology, Huangshi Central Hospital, Affiliated Hospital of Hubei Polytechnic University, Edong Healthcare Group, Huangshi, Hubei, China; ^4^ Department of Clinical Laboratory, Huangshi Central Hospital, Affiliated Hospital of Hubei Polytechnic University, Edong Healthcare Group, Huangshi, Hubei, China

**Keywords:** m6A RNA modification, gastric cancer, mRNA, ncRNA, RNA methylation

## Abstract

N6-methyladenosinen (m^6^A) methylation is a frequent RNA methylation modification that is regulated by three proteins: “writers”, “erasers”, and “readers”. The m^6^A modification regulates RNA stability and other mechanisms, including translation, cleavage, and degradation. Interestingly, recent research has linked m^6^A RNA modification to the occurrence and development of cancers, such as hepatocellular carcinoma and non-small cell lung cancer. This review summarizes the regulatory role of m^6^A RNA modification in gastric cancer (GC), including targets, the mechanisms of action, and the potential signaling pathways. Our present findings can facilitate our understanding of the significance of m^6^A RNA modification in GC.

## 1 Introduction

The most abundant form of RNA modification, N6-methyladenosinen (m^6^A) methylation, occurs at the N6-position of adenosine by recognizing the motif RRm6ACH ([G/A/U] [G/A]m6AC[U/A/C]) (1–3). The m^6^A modification regulates mRNA stability, translation, or degradation. m^6^A methylation in non-coding RNA (ncRNA), not only regulates the miRNA process but also affects the translation or degradation of lncRNA and circRNA (4–12). In addition to m^6^A modification, other frequent RNA modifications include pseudouridine (Ψ), N4-acetylcytidine (ac4C), N^1^-methyladenosine (m1A), 5-methylcytidine(m5C), and N^7^-methylguanosine (m7G) (1, 13, 14). Gene expression, biological rhythms, autophagy, apoptosis, and other biological processes are all regulated by RNA modification (3). RNA modification, like DNA methylation and histone modification, is also a type of epigenetic alteration. Recent studies have shown that epigenetics has a regulatory role in the development of Gastric cancer (GC) (15). GC is the fifth most common malignancy in humans and the third leading cause of cancer-related deaths. Clinically, the late diagnosis and poor prognosis in GC cause difficulties in the treatment. Therefore, it is very important to clarify the pathogenesis of GC. Recent studies have shown that m^6^A plays a critical role in GC formation. With the continuous emergence of m^6^A publications, there is an urgent need for a review to summarize its significance. However, we were unable to find relevant reviews in a large number of basic medical research (16–21). We will discuss the current regulatory mechanism of m^6^A methylation in the GC process, and propose the critical role of m^6^A modification in the diagnosis, treatment, and prognosis. This will provide us with a profound understanding of the m^6^A RNA modification as well as potential strategies for future clinical treatment in GC.

## 2 The m^6^A modification on RNA

### 2.1 The mechanism of m6A RNA modification on mRNA

#### 2.1.1 Methylation modification of mRNA by “writers”

**Figure 1:**
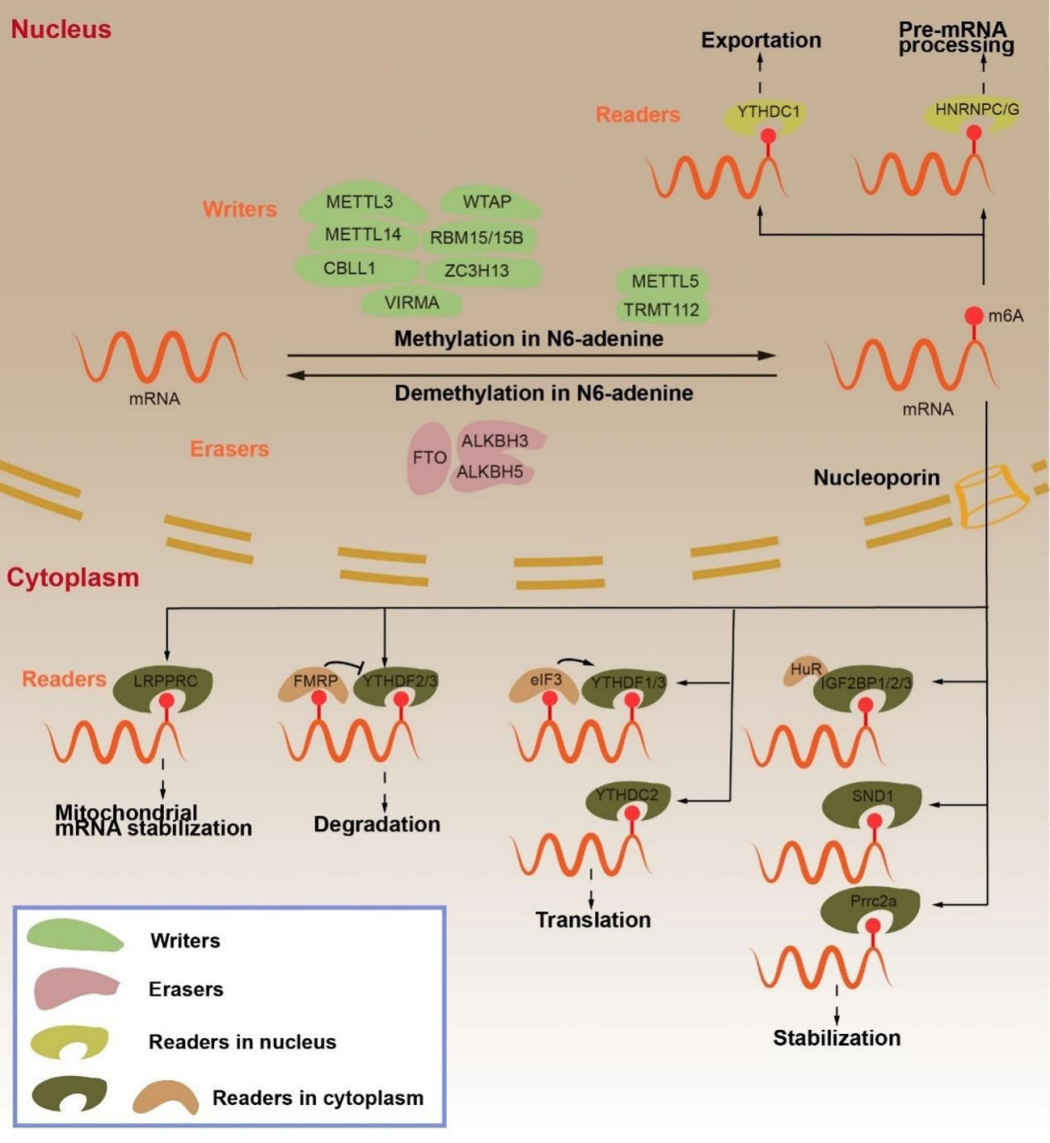
Regulation and function of m^6^A RNA modifications in mRNA by “writers”, “erasers” and “readers”. The m^6^A “writers”, including METTL3, METTL14, CBLL1, WTAP, RBM15/15B, ZC3H13 and VIRMA, cause mRNA methylation in N6-adenosine. The m^6^A “erasers”, including FTO, ALKBH3 and ALKBH5, lead to demethylation in N6-adenosine. The m^6^A “readers”, include YTHDC1, HNRNPC/G, LRPPRC, YTHDF2/3, FMRP, YTHDF1/3, YTHDC2, eIF3, IGF2BP1/2/3, SND1, Prrc2a. YTHDC1 transports mRNA from the nucleus to the cytoplasm. HNRNPC/G participates in pre-mRNA processing. LRPPRC maintains mitochondrial mRNA stabilization. YTHDF2/3 promotes mRNA degradation, but FMRP inhibits mRNA degradation. YTHDF1/3, eIF3 AND YTHDC2 promote mRNA translation. IGF2BP1/2/3, SND1, Prrc2a enhance mRNA stabilization.

m^6^A RNA modification is a dynamic reversible chemical modification that is regulated by “writers”, “erasers”, and “readers” (**Figure 1**) (8). The core components of methyltransferase complex (MTC) include methyltransferase like 3 (METTL3), methyltransferase like 14 (METTL14), and Wilms tumor 1-associating protein (WTAP), which is the main factor of “writers” in inducing m^6^A methylation (9). In the process of MTC-induced mRNA methylation, METTL3 has an S-adenosyl methionine (SAM)-binding sequence. METTL14, as a structural support of RNA binding, binds H3K36me3 and enhances the catalytic ability of METTL3 (3, 4, 22–24). METTL3 and METTL14 form heterodimers that WTAP recruits into specific RNA sequences to methylate mRNA (25–27). Intriguingly, METTL3 acts as a “reader” to recruit eIF3, thereby promoting mRNA translation in the cytoplasm (28). Furthermore, the MTC contains RNA-binding motif protein 15 and 15B (RBM15/15B), Cbl proto-oncogene like 1 (CBLL1/HAKAI), zinc-finger CCCH domain-containing protein 13 (ZC3H13), methyltransferase like 5 (METTL5), tRNA methyltransferase activator subunit 11-2 (TRMT112), and KIAA1429 (25, 29–31). KIAA1429 is the most studied “writer” among them. KIAA1429, also known as vir-like m^6^A methyltransferase-associated protein (VIRMA), mediates m^6^A methylation preferentially at the 3’ UTR and near the stop codon (32, 33).

#### 2.1.2 Methylation modification of mRNA by “erasers”

In contrast, the role of “writers” and “erasers” remove m^6^A modifications from mRNA. Fat mass and obesity-associated (FTO) and AlkB homolog 5 (ALKBH5) are both Fe^2+^ and 2-oxoglutarate (2OG)-dependent AlkB dioxygenases that mediate demethylation by recognizing the methylation sites on m^6^A and subsequently regulating the mRNA function (34, 35). The difference between the two is that FTO oxidizes m^6^A to N6-hydroxymethyladenosine (hm6A) and N6-formyladenosine (f6A) and then hydrolyzes it to adenosine, while ALKBH5 locates the nuclear speck and directly eliminates mRNA m^6^A methylation on the mRNA (24, 30, 36). ALKBH3 is like a demethylase, but it is preferable to demethylate tRNA (3, 13). “Writers” and “erasers” interact to regulate the abundance of m^6^A, while “readers”—as special RNA-binding proteins—regulate the stability, splicing, nuclear output, translation, or degradation of m^6^A-modified mRNA (37, 38).

#### 2.1.3 Methylation modification of mRNA by “readers”

“Readers” are mainly divided into three classes, YT521-B homology (YTH) domain family, heterogeneous nuclear ribonucleoproteins (hnRNPs), and insulin-like growth factor 2 mRNA-binding proteins (IGF2BPs). YTH domain family includes YTHDF1, YTHDF2, YTHDF3, YTHDC1, and YTHDC2 (10). YTHDF1 binds the eukaryotic initiation factor (eIF3) to the m^6^A site on mRNA and promotes mRNA translation. YTHDF2, on the contrary, recognizes m^6^A modification sites on the RNA 3’ UTR and recruits the CCR4-NOT deadenylase complex to promote mRNA degradation. YTHDC2 is also associated with RNA hydrolases to promote mRNA degradation (3, 24). YTHDF3 functions as an accelerator, promoting mRNA translation or degradation in collaboration with YTHDF1 or YTHDF2 (24, 34, 39–42). Intriguingly, fragile-X mental retardation protein (FMRP) has been shown to bind to YTHDF2, which inhibits the degradation of mRNA and promotes its stability (43). YTHDC1 binds the nuclear export protein serine/arginine splicing factor 3 (SRSF3) and inhibits SRSF10 in the nucleus, thereby facilitating mRNA splicing (37, 44). HNRNPs are a class of RNA-binding proteins that act as “m^6^A switches” to regulate RNA abundance. HNRNPA2B1 enhances the stability of m^6^A-modified mRNA, and both HNRNPC and HNRNPG can regulate pre-mRNA alternative splicing (23, 45–47). The IGF2BPs are the third type of “readers”. Unlike YTHDF2, IGF2BP1-3 promotes mRNA stability and translation by recruiting ELAV-like RNA-binding protein 1 (ELAVL1), which is also known as HuR (23, 38, 48). Furthermore, LRPPRC leucine-rich PPR-motif-containing protein (LRPPRC) can promote mRNA stability in the mitochondria, whereas Staphylococcal Nuclease and Tudor Domain Containing 1 (SND1) and Proline-Rich Coiled-Coil 2A (Prrc2a) such as IGF2BPs promote mRNA stability in m^6^A modification (24, 34, 49, 50).

### 2.2 m^6^A methylation interacts with ncRNA to regulate functions

**Figure 2:**
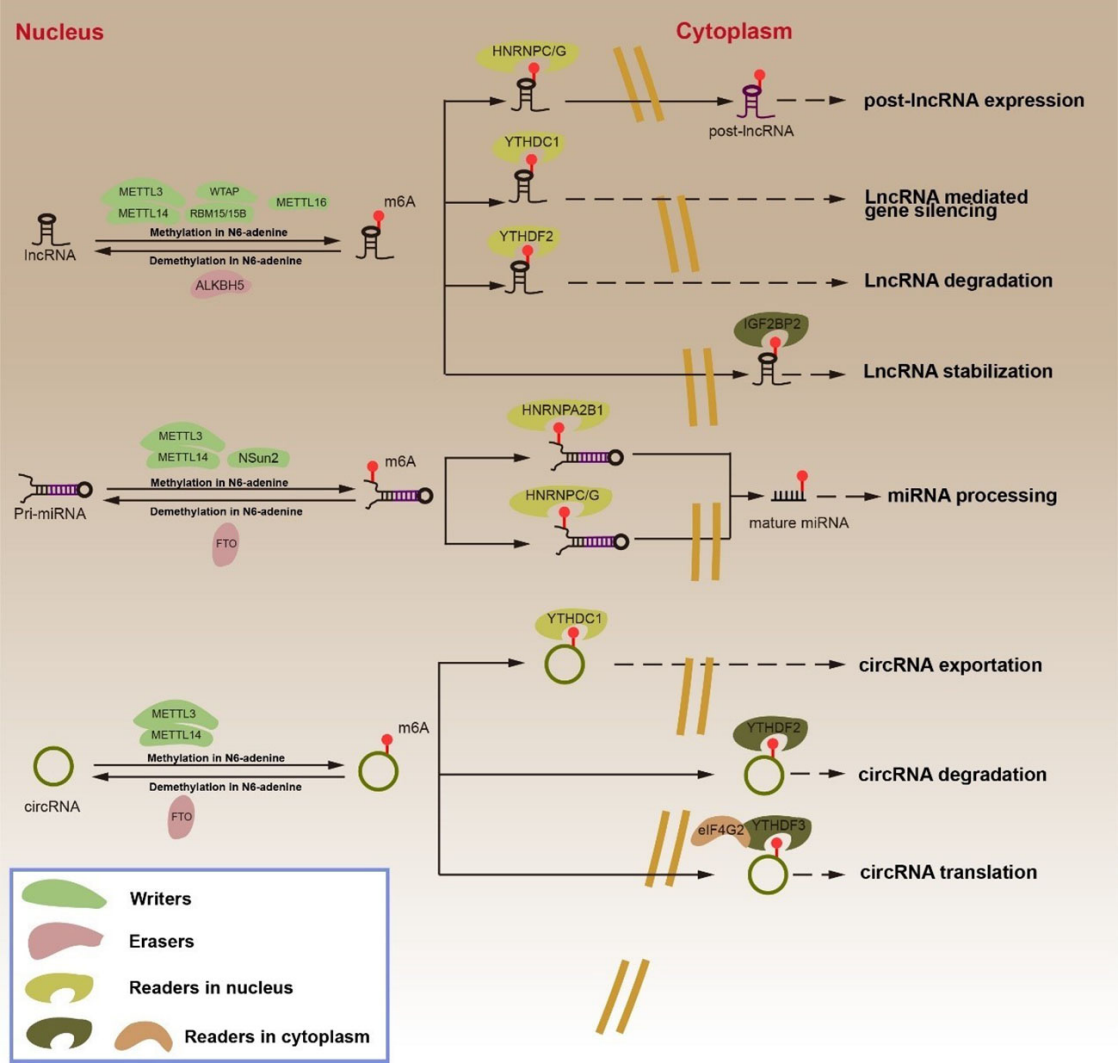
Regulation and function of m^6^A RNA modifications in ncRNA by “writers”, “erasers” and “readers”. The m^6^A “writers”, including METTL3, METTL14, WTAP, RBM15/15B and METTL16, cause lncRNA methylation in N6-adenosine. METTL3, METTL14 and NSun2 cause pri-miRNA methylation in N6-adenosine. METTL3 and METTL14 cause circRNA methylation in N6-adenosine. The m^6^A “erasers”, ALKBH5 lead to lncRNA demethylation in N6-adenosine. FTO result in pri-miRNA and circRNA demethylation in N6-adenosine. The m^6^A “readers”, HNRNPC/G promotes lncRNA expression. YTHDC1 mediates gene silencing. YTHDF2 leads to lncRNA degradation. IGF2BP2 enhances lncRNA stabilization. HNRNPA2B1 and HNPNPC/G participates in miRNA processing. YTHDC1 transports circRNA from the nucleus to the cytoplasm. YTHDF2 causes circRNA degradation. YTHDF2 and eIF4G2 facilitate circRNA translation.

In addition to mRNA, m^6^A modification modulated ncRNA splicing, maturation, and other processes (**Figure 2**) (6, 51).

#### 2.2.1 Regulation of LncRNA by m^6^A modification

LncRNA can be categorized into 5 categories in accordance with the different positions of its transcription genes: internal lncRNA (lincRNA), internal lncRNA, sense lncRNA, antisense lncRNA, and bidirectional lncRNA. Although lncRNAs lack ORF and cannot be used as templates for protein synthesis, they do play an important regulatory role in RNA transcription and post-transcriptional modification (52, 53). Past studies have demonstrated that m^6^A modification can regulate the function of lncRNA. On one hand, m^6^A modification regulates the interaction between lncRNA and specific DNA sites *via* the RNA-DNA triple helix structure; on the other hand, m^6^A modification affects the binding of “readers” to lncRNA (54–56). YTHDC1 binds m^6^A methylated long-noncoding RNA X inactivity-specific transcripts (XIST) and promotes XIST-mediated gene silencing through the modification of WTAP and RBM15/15B (57, 58). LncRNA metastasis-associated lung adenocarcinoma transcript 1 (MALAT1) positively correlates with the methylation modification of METTL3/METTL14. The binding of “m^6^A switch” HNRNPC to MALAT1 reduced with reducing expression of METTL3 or METTL14. In addition, MALAT1’s binding ability to HNRNPC/G was significantly enhanced following m^6^A methylation at A2577 of MALAT1. Both HNRNPC and HNRNPG were found to be capable of regulating MALAT1 in alternative splicing and in promoting the expression of post-lncRNA (59–61). METTL16 is a methyltransferase of U6 spliceosome small nuclear RNA (snRNA), but it directly interacts with the MALAT1 triple helix to promote the cleavage of MALAT1 (62). m^6^A-modified lncRNAs play an important function in different cancers. ALKBH5, for example, promotes tumorigenesis in GC by reversing the methylation of nuclear para-peckle assembly transcript 1 (NEAT1) (63). YTHDF2 promotes the degradation of FOXF1-adjacent noncoding developmental regulatory RNA (FENDRR) in endometrioid endometrial carcinoma (EEC) (64). The co-action of METTL3 and METTL14 with m^6^A modification promotes the stability of lncRNA LNCAROD in head and neck squamous cell carcinoma (HNSCC). The lncRNA differentiation antagonizing nonprotein coding RNA (DANCR) is essential in cancer development. IGF2BP2 has been demonstrated to recognize the m^6^A modification site on lncRNA DANCR, which promotes its stability (65).

#### 2.2.2 Regulation of miRNA by m^6^A modification

MiRNA is a single-stranded non-coding RNA of length 21–25 nucleotides. MiRNA can form the RNA-induced silencing complex (RISC), which binds the 3’ UTR of target mRNA, resulting in translational inhibition or degradation of mRNA (66–68). MiRNA processing can mainly be categorized into two phases. The initial step is for DGCR8 and DROSHA to splice pri-miRNA generated by miRNA gene transcription to form pre-miRNA. After the pre-miRNA is exported from the nucleus to the cytoplasm *via* export protein 5 (XPO5), DICER further processes the pre-miRNA into mature miRNA (66, 69–71). Intriguingly, the maturation of miRNAs relies on m^6^A modification (6). It has been reported that the maturation of pri-miRNAs is m^6^A-dependent (72). METTL3 is a common m^6^A regulatory protein that associates with DGCR8 in bladder cancer cells to promote the maturation of miR-221/222 (73). METTL3 also promotes the cleavage of pri-miRNA-let-7e and pri-miR-17-92 to form let-7e-5p and miR-18a-5p (74). METTL14 positively regulates the maturation of the tumor suppressors miR-126, miR-375, and miR-19a, as well as inhibiting invasion and migration in several cancers (75–77). METTL3 and METTL14 cooperate to promote m^6^A RNA modification, but they have been reported to demonstrate opposite effects on the occurrence of the same tumor. On one hand, although METTL3 and METTL14 form a complex to jointly promote m^6^A RNA modification, METTL3 or METTL14 in this complex can have their direct targets. This target can act as a tumor suppressor or an oncogene, and the abnormal expression of METTL3 or METTL14 biases them toward the regulation of a certain target, thereby exhibiting the opposite effect. In addition, the complex formed by METTL3 and METTL14 promotes m^6^A RNA modification, and “Readers” further process RNAs in which m^6^A occurs to determine the state of these RNAs. Therefore, in different types of same cancer, owing to the differences in the expression of different “Readers”, METTL3 or METTL14 can have opposite effects in the same cancer type. In fact, the current understanding of this mechanism is unclear, warranting further research. Similarly, the demethylase FTO may influence miRNA stability by inhibiting the expression of miR-181b-3p and miR-576 by interacting with DGCR8 (78, 79). Various “readers” proteins promote miRNA maturation by recognizing the m^6^A-modified sites in addition to “writers” in m^6^A -modified regulatory proteins. During pancreatic cancer progression, NKAP leads to the overmaturation of miR-25 under overexpressed METTL3 m^6^A modification, thereby inhibiting PHLPP2 and activating the AKT-p70S6K signaling to promote cancer progression (80). Moreover, HNRNPA2B1 can interact with DGCR8 and DROSHA to promote their binding to miRNAs (81–83). HNRNPC binds miR-21 directly to promote miRNA maturation (84). m^6^A modification, however, can also inhibit miRNA processing (6). Through methylation, NSun2 inhibits the splicing of pri-miR125b2 to pre-miR125b2, which reduces the miR-125 expression (85, 86).

#### 2.2.3 Regulation of circRNAs by m^6^A modification

CircRNAs belongs to a class of circular non-coding RNAs with a closed circular structure. CircRNAs can be classified into the following 4 types: exonic circRNAs (ecircRNAs), intronic circRNAs (ciRNAs), exon-intron circRNAs (elciRNAs), and those formed by the circularization of viral RNA, tRNA, and shRNA (87). CircRNAs, as competitive endogenous RNAs (ceRNA), can sponge miRNA. CircRNA function can be influenced by m^6^A modification. Some circRNAs have protein-coding potential, while m^6^A modification can drive the translation process. Promoter eIF4G2 and YTHDF3, under the enhanced action of methyltransferase METTL3/14, start the translation of circRNA, while demethylase FTO inhibits the translation of circRNA (56, 88). CircCPSF6 is recognized and degraded by YTHDF2 during ALKBH5 demethylation (89). YTHDC1 has been reported to promote the nuclear output of circRNA NOP2/Sun RNA methyltransferase 2 (circNSUN2) (88).

## 3 The regulatory mechanism of m^6^A in GC

By regulating protein synthesis and changes in the signaling pathways, the m^6^A modification induces cancer progression, such as protective autophagy, metastasis, abnormal proliferation, EMT, drug resistance, and abnormal glycolysis in cancer cells. The abnormally expressed m^6^A regulators can be utilized as cancer therapeutic drug targets to expand cancer diagnosis and treatment options (3, 20, 90, 91). **Table 1** summarizes the regulatory effects of m^6^A methylation on the common pathways to GC.

**Table 1:** Regulation of the signaling pathway by m^6^A RNA modification in GC.

Gastric cancer cell line	m^6^A regulators	Type of regulation	Target	Mode of action	Function	Targeted mRNA/pathways	Ref.
**AGS, MKN45**	METTL3 (Writers)	oncogene	N/A	N/A	promotes tumor proliferation inhibits tumor apoptosis	METTL3—BAX, caspase-3, BCL-2 METTL3—p-AKT—P70s6k, Cyclin D1	(92)
**BGC823, AGS**	METTL3 (Writers), IGF2BP3 (Readers)	oncogene suppressor	HDGF	Stabilization	promotes glycolysis and tumor angiogenesis	METTL3—IGF2BP3—HDGF—GLUT4, ENO2	(93)
**BGC823, AGS**	METTL3, HUR (Writers)	oncogene	ZMYM1	N/A	promotes EMT and metastasis	METTL3—ZMYM1—E-cadherin	(94)
**HGC27, MGC803**	METTL14 (Writers) FTO (Erasers)	oncogene suppressor	N/A	N/A	inhibits the proliferation, migration, and invasion of gastric	METTL14, FTO—Wnt, PI3K—AKT	(95)
**SGC7901, MGC803**	KIAA1429 (Writers)	oncogene	c-JUN	Stabilization	promotes GC proliferation	KIAA1429—c-JUN	(96)
**AGS**	METTL3 (Writers)	oncogene	SOCS2	N/A	promotes GC proliferation	METTL3—SOCS2	(97)
**HGC27, MGC803**	METTL3 (Writers)	oncogene	BATF2	N/A	promotes growth and metastasis of GC	METTL3—BATF2—P53—ERK	(98)
**SGC7901, AGS**	METTL3 (Writers)	oncogene	MYC	Stabilization	promotes growth and metastasis of GC	METTL3—MYC—MCM5, MCM6	(99)
**SGC7901**	WTAP (Writers)	oncogene	HK2	Stabilization	promotes GC proliferation and glycolysis	WTAP—HK2	(21)
**Gastric cancer cell line**	**m^6^A regulators**	**Type of regulation**	**Target**	**Mode of action**	**Function**	**Targeted mRNA/pathways**	**Ref.**
**SGC7901**	METTL14 (Writers)	suppressor	N/A	N/A	inhibits the proliferation, migration, invasion and EMT of GC	METTL14—PI3K—AKT	(100)
**HGC27, MGC803**	YTHDF1 (Readers)	oncogene	FZD7	Translation	promotes gastric carcinogenesis by activating the Wnt pathway	YTHDF1—FZD7—β-catenin	(101)
**N/A**	FTO (Erasers)	suppressor	mTORC1	Demethylation	inhibits autophagy and promotes drug resistance of GC	FTO—mTORC1	(102)
**BGC823, AGS**	METTL3 (Writer)	oncogene	Snail	N/A	promotes EMT and metastasis of GC	HOXA10—TGF-β/Smad—METTL3—snail	(103)
**AGS, MKN45**	FTO (Erasers)	oncogene	HOXB13	Demethylation	promotes GC cell migration and invasion	FTO—HOXB13—PI3K—AKT—mTOR	(104)
**AGS, MKN45**	METTL3 (Writer)	oncogene	YAP	N/A	promotes proliferation and migration of GC	METTL3—YAP	(105)
**SGC-7901, MKN45**	METTL3 (Writer)	oncogene	SPHK2	Translation	facilitates GC cell proliferation, migration and invasion	METTL3—SPHK2—KLF2	(106)
**AGS, MGC803**	METTL16 (Writers)	oncogene	cyclinD1	Stabilization	facilitates GC cell proliferation	METTL16—cyclin D1	(107)
**BGC823, MGC803**	FTO (Erasers)	oncogene	ITGB1	Demethylation	promotes GC metastasis	FTO—ITGB1—p-FAK	(108)
**Gastric cancer cell line**	**m^6^A regulators**	**Type of regulation**	**Target**	**Mode of action**	**Function**	**Targeted mRNA/pathways**	**Ref.**
**AGS, BGC823**	YTHDF1	oncogene	USP14	Translation	facilitates the tumorigenesis and metastasis of GC	YTHDF1—USP14	(109)
**AGS, HGC27**	YTHDC2	N/A	YAP	Translation	promotes proliferation and migration of GC	YTHDC2—YAP	(110)
**AGS**	YTHDF2	suppressor	FOXC2	Degradation	inhibits the proliferation, invasion, and migration of GC	YTHDF2—FOXC2	(111)
**SGC7901, BGC823**	ALKBH5 (Erasers)	oncogene	lncRNA NEAT1	Demethylation	promotes GC invasion and metastasis	ALKBH5—lncRNA NEAT1— EZH2	(63)
**SGC7901, BGC823**	METTL3 (Writers)	oncogene	ARHGAP5	Stabilization	promotes chemoresistance in GC	LncRNA ARHGAP5-AS1—METTL3—ARHGAP5	(112)
**HGC27**	METTL3(Writer), IGF2BP1(Reader)	oncogene	SEC62	Stabilization	prevents GC progression	miR-4429—METTL3— IGF2BP1—SEC62	(113)
**AGS, MKN45**	METTL3 (Writer)	oncogene	miR-1792	Processing	promotes sensitivity to everolimus in GC	METTL3—miR-1792—PTEN, TMEM127—AKT—mTOR	(114)
**MGC803, MKN45**	ALKBH5 (Erasers)	oncogene	Nanog	Demethylation	promotes GC proliferation	LncRNA NRON—ALKBH5—Nanog	(115)
**SGC7901, BGC823**	KIAA1429 (Writers)	oncogene	LINC00958	N/A	promotes glycolysis and tumor angiogenesis of GC	KIAA1429—LINC00958—GLUT1	(116)
**Gastric cancer cell line**	**m^6^A regulators**	**Type of regulation**	**Target**	**Mode of action**	**Function**	**Targeted mRNA/pathways**	**Ref.**
**HGC27, MGC803**	METTL3 (Writer)	oncogene	CDCP1	Translation	promotes proliferation and invasion of GC	EEDmiR-338-5p—METTL3—CDCP1	(117)
**AGS, HGC27**	METTL14 (Writer)	oncogene	LINC01320	N/A	promotes the proliferation, migration, and invasion of GC	METTL14—LINC01320—miR-495-5p—RAB19	(118)
**MGC803, AGS**	N/A	suppressor	N/A	N/A	inhibits the proliferation of GC	miR-660—E2F3	(119)

AKT, protein kinase B; BATF2, Basic Leucine Zipper ATF-Like Transcription Factor 2; CDCP1, CUB Domain Containing Protein 1; DDIT3, DNA damage inducible transcript 3; EED, embryonic ectoderm development protein; eIF3a, eukaryotic translation initiation factor 3 subunit a; ERK, extracellular regulated protein kinases; EMT, epithelial–mesenchymal transition; ENO2, Enolase 2; E2F3, E2F Transcription Factor 3; FAK, focal adhesion kinase; FZD7, Frizzled7; GLUT4, glucose transporter 4; HMGA2, High Mobility Group AT-Hook 2; HBXIP, hepatitis B X-interacting protein; HDGF, Hepatoma-derived growth factor; HK2, hexokinase 2; HOXB13, Homeobox B13; HOXA10, Homeobox A10; H3K27me, histone H3 lysine-27 trimethylation 3; IGF1R, Insulin-like Growth Factor 1 Receptor; JAK, Janus kinase; MCM5/6, Minichromosome Maintenance Complex Component 5/6; KLF2, Kruppel Like Factor 2; LATS1/2, large tumor suppressor 1 and 2; LEF, lymphoid enhancer-binding factor; MST1/2, mammalian sterile 20-like kinases 1 and 2; MYC, MYC Proto-Oncogene; N/A, Not applicable; PI3K, phosphatidylinositide 3-kinases; PTEN, phosphatase and tensin homolog; P70S6K, p70 Ribosomal Protein S6 Kinase; SAV1, salvador homolog 1; SAM, S—adenosyl methionine; SEC62, SEC62 homolog preprotein translocation factor; SOCS, Suppressor of Cytokine; SPHK2, Sphingosine kinase-2; STAT, signal transducers and activators of transcription; TAZ, transcriptional co-activator with PDZ-binding motif; TCF, T-cell factor; TCGA, The Cancer Genome Atlas; TEAD, Transcriptional enhanced associate domain; TGF-β, Transforming Growth Factor-β; ULK1, Unc-51 Like Autophagy Activating Kinase 1; USP14, Ubiquitin Specific Peptidase 14; VEGF, vascular endothelial growth factor; YAP, Yes-associated protein; ZMYM1, zinc finger MYM-type containing 1.

### 3.1 Regulation of m^6^A mRNA modification in GC

#### 3.1.1 The m^6^A modification promotes oncogene expression and leads to abnormal proliferation in GC

**Figure 3:**
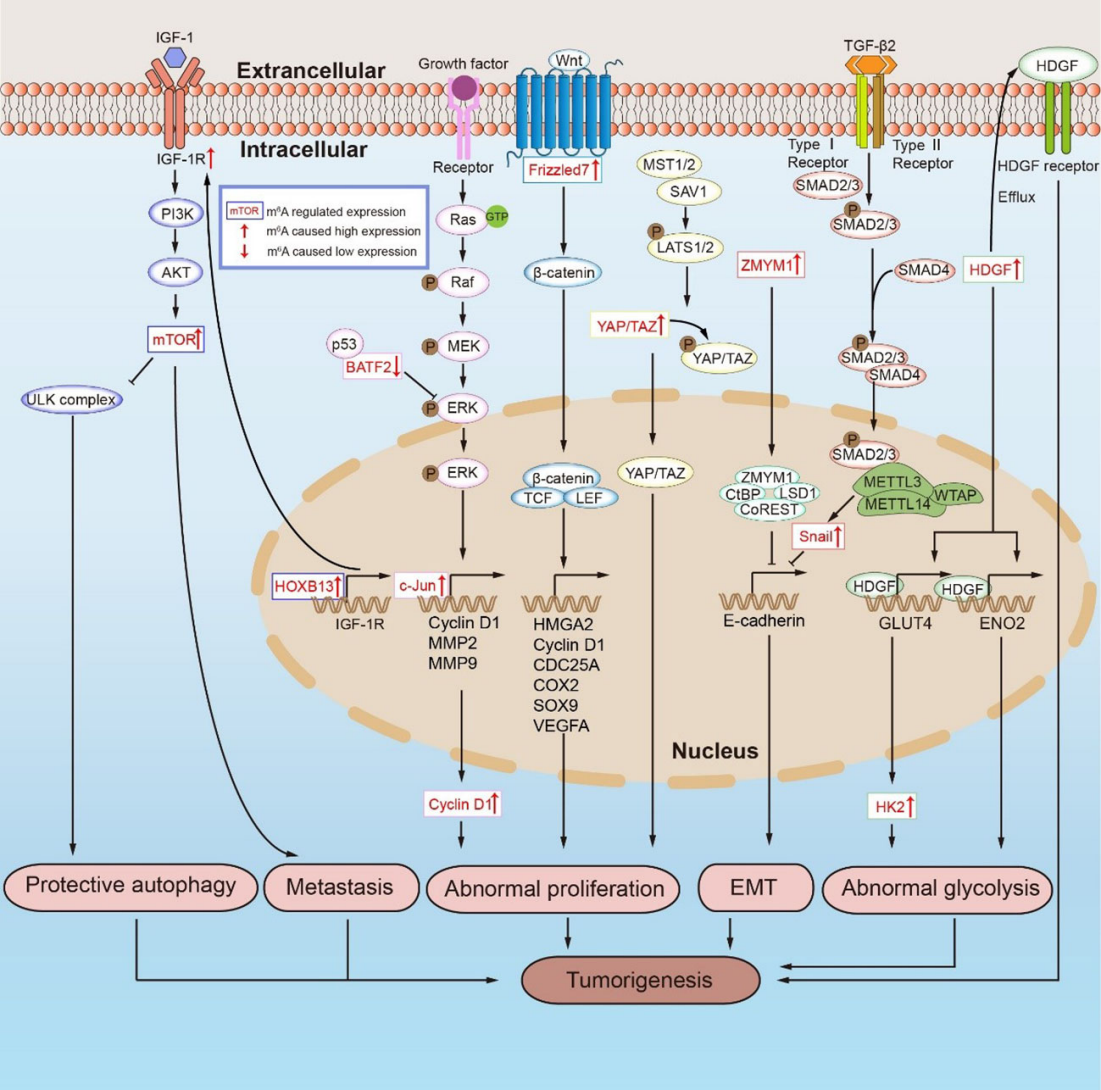
m^6^A RNA modifications mediate tumorigenesis in GC by regulating mRNA expression. PI3K/AKT/mTOR signaling pathway promotes protective autophagy and metastasis in GC. Ras/Raf/MAPK signaling pathway, Hippo signaling pathway and Wnt/β-catenin signaling pathway promote the abnormal proliferation of GC cells. The upregulation of ZMYM1 inhibits the expression of E-cadherin, which lead to the occurrence of EMT in GC. HDGF, including karyotype HDGF and secreted HDGF, cause abnormal glycolysis and tumorigenesis in GC.

Cancer progression is related to abnormal tumor proliferation, which is regulated by m^6^A modification. Ras is activated when a growth factor binds to the receptor, which in turn binds GTP to form Ras-GTP. The activated Ras affects multiple downstream proteins, including Ras-GTP, which then recruits and leads to Raf phosphorylation activation. The activated Raf kinase then binds to and activates the downstream MEK and ERK. After the phosphorylation-activated ERK is transferred from the cytoplasm to the nucleus, it can promote the phosphorylation activation of transcription factors such as c-Jun, MYC, and SP1, thereby inducing the expression of cell cycle and proliferation genes (**Figure 3**) (120, 121). BATF2 is a tumor suppressor gene in GC that inhibits the ERK phosphorylation by binding with P53, while METTL3 methylation inhibits the BATF2 expression (98, 122, 123). In addition, KIAA1429 directly targets and promotes the expression of c-Jun, facilitates the transcription of downstream MMP3, MMP9, and cyclin D1, and causes GC abnormal proliferation (**Figure 3**) (96, 98). Notably, METTL16 can promote the stability and high expression of cyclin D1 mRNA in this signaling pathway *via* m^6^A methylation, but whether cyclin D1 is the direct target of METTL16 requires further research (107). METTL3 promotes the MYC expression by m^6^A methylation and positively regulates the key components of the MYC pathway MCM5 and MCM6. Intriguingly, silencing HBXIP effectively suppressed the process, although further studies on the downstream targets of the HBXIP/METTL3/MYC axis are needed (1, 99). Similarly, the Wnt/β-catenin signaling pathway regulates abnormal GC proliferation (**Figure 3**) (124). YTHDF1 can promote the translation of FZD7, a key molecule of the Wnt signaling pathway. FZD7 binds to the Wnt protein on the cell membrane, which promotes downstream β-catenin to enter the nucleus from the cytoplasm. β-catenin is a multifunctional protein that can not only act as an adaptor protein in the cytoplasm to promote cell adhesion but also bind to the transcription factor TCF/LEF in the nucleus to promote the transcription of downstream target genes HMGA2, cyclin D, CDC25A, COX2, Sox9, and VEGFA (101, 125–127). Therefore, YTHDF1 overexpression significantly promotes the activation of the Wnt pathway (101). Interestingly, YAP/TAZ regulates β-catenin in the Wnt pathway. YAP/TAZ can form the β-catenin destruction complex in the cytoplasm, resulting in β-catenin degradation. In the nucleus, YAP binds β-catenin to form a complex that promotes the expression of downstream target genes (128–130). An active Hippo signaling pathway results in MST1/2 and SAV1 phosphorylation activation of LATS1/2. Activated LATS1/2 further phosphorylates YAP/TAZ, preventing it from entering the nucleus, and therefore blocking the regulation of downstream target genes expression when the transcriptional co-activators YAP/TAZ link to TEAD (**Figure 3**) (105, 110, 131–133). m^6^A methylation promotes abnormal proliferation in GC by regulating the expression of YAP in the Hippo signaling pathway. METTL3 induces methylation of YAP1 mRNA, and YTHDC2 recognizes m^6^A methylated YAP mRNA and promotes its translation (105, 110). The expression of METTL3 is positively correlated with the phosphorylation level of AKT. The downregulation of METTL3 resulted in low levels of p70S6K and cyclinD1, both downstream AKT (92). When the *METTL3* is silenced, SOCS2 is unable to activate the JAK/STAT signal pathway, thereby inhibiting the proliferation, metastasis, and apoptosis in GC cells (97). Although YTHDF2 degradation of FOXC2 mRNA can inhibit the proliferation of abnormal GC, the upstream regulators in the YTHDF2/FOXC2 pathway have not been explored (111). m^6^A modification is implicated in several signaling pathways and mediates abnormal proliferation in GC. This finding suggests that m^6^A-modified mRNA in the regulation of abnormal proliferation is a potential therapeutic target with significant clinical implications.

#### 3.1.2 m^6^A modification promotes the metastasis of GC

The progression of cancer is marked by distant metastasis of tumor cells. The PI3K/AKT/mTOR signaling pathway is often activated during tumorigenesis. This pathway not only regulates cell proliferation, apoptosis, autophagy, and other physiological processes but is also closely related to cancer cell metastasis (134, 135). METTL14 overexpression has been reported in studies to inhibit the PI3K/AKT/mTOR pathway activation and metastasis in GC (**Figure 3**) (100). METTL3 cooperates with YTHDF1 to promote SPHK2 mRNA translation, while m^6^A modification promotes GC metastasis *via* the METTL3/SPHK2/KLF2 axis, suggesting that the regulatory process is related to the poor prognosis in GC (106, 136). USP14 is a member of the USP ubiquitinase subfamily, and its expression in the GC tissues is higher than that in normal tissues. By knocking down USP14, it was found that the decreased expression of USP14 made GC cells sensitive to cisplatin by blocking the Akt or ERK signaling pathways. YTHDF1 could promote the translation of USP14 protein, which then promoted the tumorigenesis and metastasis of GC (109, 137). The PI3K/AKT/mTOR signaling pathway is activated in GC by HOXB13 overexpression, which is upregulated by FTO. In addition to “writers” and “readers”, the regulation of “erasers” in GC is equally significant. Past studies have shown that HOXB13 is highly expressed in GC cells due to increased demethylation of FTO and activation of the PI3K/AKT/mTOR signaling pathway (**Figure 3**). Further research revealed that HOXB13 enhanced the expression of IGF-1R by acting on the promoter region of IGF-1R. The specific mechanism of HOXB13 controlling the IGF-1R expression warrants further analysis, although it is suggested that the FTO/HOXB13/IGF-1R/PI3K/Akt/mTOR axis should be explored as a potential therapeutic target for GC (104). FTO induces ITGB1 mRNA hypomethylation and promotes its expression in the FTO/ITGB1/p-FAK axis. Since ITGFB1 is a key signaling molecule in the signal transduction between cells and the extracellular matrix, increased ITGFB1 expression promotes metastasis in GC (108, 138, 139). Although METTL3 mediates PI3K/AKT/mTOR pathway activation, its direct target remains unknown. Therefore, positively searching for the downstream effectors of m^6^A regulatory proteins in the PI3K/AKT/mTOR signaling pathway can facilitate a better understanding of the role of this pathway in GC metastasis.

#### 3.1.3 m^6^A modification regulates the expression of key enzymes in the Warburg effect to promote abnormal glycolysis

Abnormal glycolysis is one of the hallmarks of further deterioration in GC. Glycolysis is an important energy source for GC deterioration in the metabolic process (140–143). Only HDGF was always regulated by METTL3 in four GC cell lines with an overexpression or knockout of METTL3, while the expression of GLUT4 and ENO2 increased correspondingly in GC cells with a high expression of METTL3 (**Figure 3**) (93). HDGF is essential in GC metastasis. The karyotype HDGF functions act as a transcriptional regulator to accelerate its expression in the promoter regions of GLUT4 and ENO2. The secreted HDGF promotes tumor angiogenesis by inducing VEGF (93, 144–146). The functional acetylation of H3K27me3 by p300 activates METTL3, which then promotes m^6^A methylation of HDGF mRNA. On the other hand, IGF2BP3 recognition of the m^6^A modification sites increases HDGF mRNA stability. HDGF promotes the expression of GLUT and ENO2 in the nucleus and initiates the glycolytic pathway in tumor cells (93). WTAP stabilized HK2 mRNA by binding to the 3’-UTR m^6^A site of HK2 mRNA and promoted its expression, making WTAP a novel target for GC therapy (21, 147). Moreover, ENO2 participates in the Warburg effect and together promotes aberrant glycolysis in GC under the regulation of m^6^A modification (93). We discovered that blocking tumorigenesis in the glycolysis process in GC can be employed as a new clinical treatment, and the proteins regulated by WTAP or those involved in m6A modification can be new drug targets.

#### 3.1.4 m^6^A modification regulates E-cadherin protein expression to affect the EMT process in GC

In GC, EMT is the key step influencing distant metastasis (94). E-cadherin is a cell adhesion molecule that can promote adhesion between cells or between cells and the intercellular matrix. The emergence of EMT is marked by the deletion of the epithelial marker E-cadherin and the synthesis of the mesenchymal markers vimentin, N-cadherin, and smooth muscle actin (αSMA), which mark the occurrence of EMT (148). Past studies have demonstrated that METTL3 modifies ZMYM1 mRNA and promotes its high expression. ZMYM1 recruits the CtBP/LSD1/CoREST complex and inhibits the role of the E-cadherin promoter, resulting in EMT in GC (**Figure 3**) (94). Snail is a zinc finger protein that inhibits the expression of E-cadherin (149). After modifying the snail protein *via* the HOXA10/TGF-β/Smad/METTL3/snail signaling pathway, METTL3 inhibited the E-cadherin expression, thereby facilitating GC metastasis (**Figure 3**) (103). In another study, METTL14 and FTO knockdown activated the Wnt/PI3K-AKT signaling pathways (**Figure 3**) (95). Wnt/PI3K-AKT pathways promote EMT in GC by inhibiting the expression of E-cadherin (95, 150, 151). However, it remains unclear whether m^6^A modification inhibits E-cadherin expression through pathways besides the snail pathway and Wnt/PI3K-AKT signaling pathway toward promoting the EMT generation.

#### 3.1.5 m^6^A modification promotes protective autophagy in GC by PI3K/AKT/mTOR axis

Autophagy is a double-edged sword in tumorigenesis. On one hand, autophagy inhibits tumor growth by eliminating harmful cellular components and preventing DNA damage. Autophagy, on the other hand, can provide nutrition for tumor cells while also promoting cancer progression (152). The protective autophagy of tumor cells diminishes their drug sensitivity, which contributes to cancer progression. Since the PI3K/AKT/mTOR pathway is involved in the mechanism of autophagy, targeted modulation of this pathway has become an important approach for cancer therapy (153). The FTO expression was negatively correlated with disease-free survival in GC, and it not only regulated HOXB13 in the PI3K/AKT/mTOR signaling pathway but was also related to the mTORC1 expression (**Figure 3**). When the ULK1 complex is activated, it directly phosphorylates Beclin-1 and ATG14L, thereby phosphorylating the downstream substrate VPS34 complex and promoting autophagy. mTORC1 functions in the general pathway by inhibiting the ULK1 complex, which suppresses cytoprotective autophagy and improves tumor drug sensitivity (102, 104, 152, 154–156). In the drug resistance study of GC cells, omeprazole inhibition of FTO could improve the inhibitory effect of 5-Fu, DDP, and TAX on GC cells as well as enhance the ability of mTORC1 to inhibit autophagy and improve the anti-tumor ability (102).

#### 3.1.6 m^6^A RNA modification participates in GC immune infiltration and apoptosis

m^6^A modifications were involved in regulating other functions during GC progression. WTAP is an m^6^A-modified regulatory protein, and its high expression is closely related to m^6^A methylation. Interestingly, reduced WTAP expression has been linked to T lymphocyte infiltration (157). Apoptosis is a type of programmed cell death helpful to eliminate tumor cells. METTL3 reduces the expression of anti-apoptotic protein Bcl-2 through m^6^A methylation modification, while the pro-apoptotic protein Bax is relatively increased, which then activates Caspase-3 to promote the apoptotic pathway in GC (**Figure 3**) (92).

### 3.2 Modification of ncRNA by m^6^A in GC

#### 3.2.1 Interaction between lncRNAs and m^6^A methylation during GC progression

**Figure 4:**
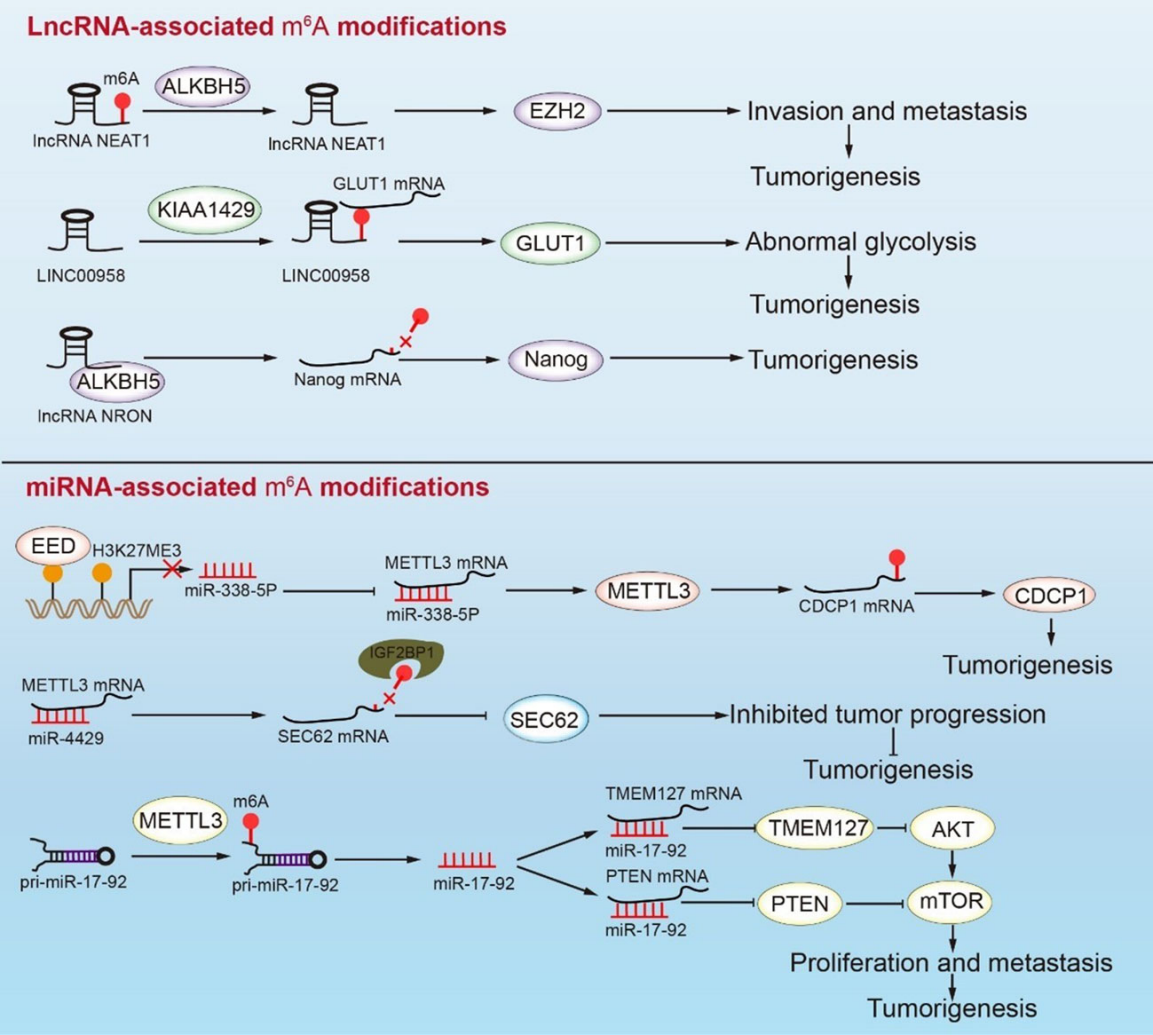
Interactions between m^6^A RNA modifications and ncRNAs mediate tumorigenesis in GC. The demethylation of NEAT1 enhances the expression of EZH2, which leads to invasion and metastasis in GC. KIAA1429 causes the methylation of LINC00958, which promotes GC abnormal glycolysis by facilitating the expression of GLUT1 mRNA. In addition, NRON binding ALKBH5 causes the demethylation of Nanog mRNA and enhances Nanog expression. The transcriptional repression of miR-338-5p rescues METTL3 mRNA, which enhances the expression of CDCP1 and leads to tumorigenesis. Conversely, miR-4429 inhibits the expression of METTL3, thus decreasing the methylation of SEC62 mRNA, which represses the SEC62 levels and inhibits tumor progression. METTL3 participates in miR-17-92 processing, which regulates the PI3K/AKT signaling pathway to promote the proliferation and metastasis of GC cells.

During tumorigenesis, m^6^A methylation interacts with lncRNAs to regulate mRNA expression. Methyltransferase KIAA1429 inhibits the decay of lncRNA LINC00958, which can target GLUT1 to mediate aerobic glycolysis in GC. Therefore, KIAA1429 accelerates the glycolysis process by modifying LINC00958 with m^6^A methylation (116). ALKBH5 mediates lncNEAT1 demethylation, which affects the combination of lncNEAT1 and EZH2, thereby promoting GC development (63). METTL14 methylation enhanced LINC01320, which then absorbed miR-495-5p like a sponge. METTL3 promotes the RAB19 expression *via* the miR-495-5p/RAB19 axis, resulting in GC proliferation, migration, and invasion. However, the LINC01320/miR-495-5p/RAB19 axis in GC has not been thoroughly investigated, and this pathway warrants additional investigation (118). LncRNAs are not only modified by m^6^A, but they may also promote or inhibit m^6^A modification. As an oncogene, LINC00470 interacts with METTL3 and mediates the PTEN mRNA degradation in a YTHDF2-dependent manner (**Figure 4**) (158). LncRNA NRON combined with ALKBH5 mediates Nanog mRNA demethylation of Nanog mRNA and promotes Nanog stability during tumorigenesis (**Figure 4**) (115). LncRNA ARHGAP5-AS1 is also related to m^6^A regulatory protein. METTL3 combined with lncRNA ARHGAP5-AS1 to promote ARHGAP5 mRNA translation and induce protective autophagy in GC cells (**Figure 4**) (112). In general, a high expression of m^6^A-modified lncRNA has been closely related to poor prognosis for GC, although the specific mechanism of lncRNA and m^6^A modification remains unclear. This observation emphasizes the need for additional research on m^6^A-modified lncRNAs.

#### 3.2.2 Interaction between m^6^A modification and miRNA

The interaction between miRNA and m^6^A-regulatory protein promotes proliferation, metastasis, and invasion in GC (**Figure 4**). Mature miR-17-92 inhibits the AKT/mTOR signaling pathway by affecting the expression of PTEN and TMEM127, which limits tumor proliferation and metastasis (114). METTL3 may promote pri-miR-17-92 processing through methylation, which is closely related to the poor prognosis in GC. The miR-660/E2F3 pathway is critical in the development of GC (119). He et al. reported that the m^6^A-modified tumor suppressor gene miR-660 could inhibit GC proliferation. Furthermore, miRNA is involved in the regulation of m^6^A-regulatory proteins. Both miR-338-5p and miR-4429 bind to METTL3 mRNA and inhibit its expression. SEC62 has a carcinogenic effect and is abundant in GC cells. It was discovered that METTL3 might interact with SEC62 mRNA *via* m^6^A modification, thereby allowing IGFBP1 to recognize and promote the stability of SEC62 mRNA. MiR-4429 inhibits the m^6^A methylation level of SEC62 mRNA by targeting METTL3, thus preventing GC progression (113). High EED expression in GC patients has been associated with poor prognosis. EED inhibited the splicing of METTL3 mRNA by downregulating the expression of miR-338-5p through H3K27me3. The increased METTL3 directly promoted the methylation modification and expression of CDCP1 mRNA, which, in turn, accelerated the occurrence in GC (117, 159). Some m^6^A regulatory proteins can precisely target specific miRNA sites, which can be employed as highly precise intervention targets. miRNAs bind to the mRNA of m^6^A regulatory proteins to regulate the m^6^A level of downstream signaling factors and provide a biomarker for future research.

#### 3.2.3 Interaction between circRNAs and m^6^A methylation during GC progression

It is well known that circRNA usually acts as ceRNA to competitively inhibit miRNA function. In GC cells, miR-30c-2-3p can inhibit the proliferation and metastasis of GC. Knocking down MTEEL14 reduced the m^6^A methylation level of circORC5, and increased the expression of circORC5, thereby reducing the content of miR-30c-2-3p (160). From this, we can know that METTL14 regulates miR-30c-2-3p/AKT1S1 axis through methylation modification of circORC5, thereby inhibiting the deterioration of GC. However, research on the connection of circRNA and m^6^A regulated is still needed.

## 4 Future prospects

### 4.1 The role of m^6^A modification in diagnosis

At the molecular level, m^6^A modification affects the stability and degradation of RNA, thereby inducing changes in protein translation and regulating signal pathways. It has been reported that the m^6^A-regulatory protein with high specificity and sensitivity Our revised content of Q1(2) by reviewer 1is crucial in GC diagnosis. YTHDF1 is highly expressed in high-risk subtypes of GC patients. By constructing a diagnostic-m6A-score (DMS) and analyzing the ROC curve, DMS was found to demonstrate high specificity and sensitivity in the diagnosis of GC (area under the ROC curve [AUC] = 0.986). The AUC, sensitivity, and specificity of YTHDF1 were 0.52, 97.9, and 8.2%, respectively, in the TCGA cohort (161). Moreover, Kaplan–Meier survival analysis revealed that GC patients with a high YTHDF1 expression had a poor overall survival (OS) rate (P = 0.0352) and a higher risk of tumor recurrence. Therefore, detecting the level of m^6^A-methylated reading protein YTHDF1 in gastric cancer tissues after treatment can serve as an indicator to predict the prognosis of patients (162). Jing et al. (163) discovered that FTO was related to the tumor stage by analyzing the expression of the m^6^A-regulatory protein gene in the prognosis for GC by utilizing UALCAN and Oncomine web resources. Wang et al. (108) analyzed the prognostic value of 33 m^6^A RNA-methylation regulators in gastric cancer through univariate and multivariate Cox regression analyses. FTO was considered an independent risk factor for predicting the poor prognosis (HR = 1.60, P = 0.027, 95% CI: 1.10–2.50), with FTO serving as the key prognostic risk factor for OS of gastric cancer patients.

### 4.2 M^6^A modification and therapy

The regulation of m^6^A RNA modification on cancer progression is helpful for the development of targeted therapeutic drugs. Targeted drugs specifically interfere with the growth, proliferation, and diffusion of tumor cells by targeting specific proteins or genes. However, with the widespread use of targeted drugs, tumor cells gradually develop drug resistance (12). By regulating FOXO3-mediated autophagy, m^6^A methylation regulates the drug resistance to sorafenib in liver cancer (164). After autophagy damage caused by LncRNA ARHGAP5-AS1, METTL3 combined with LncRNA ARHGAP5-AS1 to promote the massive expression of ARHGAP5, resulting in GC drug resistance (112). Everolimus is an mTOR inhibitor that targets PTEN and TMEM127, and the resultant inhibitory effect is more pronounced in cancers with high METTL3 expression, albeit small molecule inhibitors of METTL3 are currently under investigation (114). Furthermore, the FTO inhibitors Rhein, MA2, and R-2HG all exhibit anticancer potential. Omeprazole can reverse the inhibitory effect of FTO on mTORC1 and its downstream signaling factor DDIT3, thereby promoting the regulation of autophagy levels (102). Past studies have demonstrated that the expression level of FTO in HER2-positive breast cancer tissues is higher than that in non-tumor tissues. Among them, FTO promotes the metastasis and invasion of cancer cells through the FTO/miR-181b-3p/ARL5B signaling pathway (78). In GC, FTO promotes GC cell migration and invasion by targeting HOXB13 and ITGB1. Therefore, it presents a new research direction in the area of GC treatment to detect whether FTO is related to the treatment of HER2-positive GC in the two signaling pathways of FTO-HOXB13-PI3K-AKT-mTOR and FTO-ITGB1-p-FAK (104, 108). Microsatellite instability (MSI) is a molecular indicator of defective DNA mismatch repair (MMR). Wang et al. (165) suggested that, in the interaction of m^6^A modification with lncRNA, the m^6^A methylation level is closely related to the MSI of GC. Among them, the prognostic effect of MSI-positive GC after chemotherapy was better, and the occurrence of high MSI-H was greater in a group with a high m^6^A methylation level. Therefore, the interaction between m^6^A modification and lncRNA is a novel research direction for the treatment of MSI-positive GC (166). When compared with chemotherapy and targeted therapy, immunotherapy not only improves the living environment of patients with advanced cancer but also avoids drug resistance (167). As a result, elucidating the regulatory role of m^6^A in the diagnosis, treatment, and prognosis of GC can enable GC patients to achieve a longer survival time. Immune cells in the tumor microenvironment (TME) play a key role in tumor progression, immune escape, and their impact on immunotherapeutic response. Immune checkpoint block (ICB) therapy eliminates cancer cells by targeting programmed death molecule 1 and its ligand (PD-1/PD-L1) in cytotoxic T cells and activating the immune system (168). M^6^A modification is related to PD1/PD-L1 immunotherapy. Through the construction of the m^6^A-scoring system, it was found that m^6^A methylation modification was significantly related to tumor response to PD-1/L1 immunotherapy (20). Overexpression of FTO promotes tumor cell growth, while knockdown of FTO significantly increases tumor cells’ response to INF-γ and anti PD-L1 treatment sensitivity. The possible mechanism is that FTO reduces the levels of m^6^A in PD-1 and prevents YTHDF2-mediated PD-1 RNA decay. In addition, *in vitro* experiments revealed that blocking YTHDF1 improved the therapeutic effect of anti-PD-L1 in GC cells, and the knockout of m^6^A demethylase AlKBH5 makes tumor sensitive to anti PD-1 immunotherapy and changes immune cell recruitment (167, 169). In order to improve the efficacy of immunotherapy, it is urgent to understand the tumor microenvironment (TME) and determine the mechanism behind the low response rate of ICB. Knocking down YTHDF1 in DC cells can enhance the cross presentation of tumor antigen and the cross start of CD8+T cells. In TME, with the increase of demethylase FTO and ALKBH5, the proportion of CD4^+^ positive T cells increased gradually. This observation demonstrated that m^6^A modification participates in the regulation of TME formation and plays an active role in tumor immunotherapy. However, in the study of the effect of m^6^A RNA modification on gastric cancer, due to the different m^6^A regulatory proteins, the effects are also different, albeit the specific regulatory targets required further research. At the epigenetic level, there is further scope for research on the generation, deterioration, or treatment of cancer.

## Conclusion

m^6^A methylation, as an important post-transcriptional modification, has gradually become an important regulatory mechanism in the occurrence and development of cancer. Functionally, m^6^A RNA modification causes a change in the RNA state, which results in the abnormal expression of various proteins in cancer. We found that m^6^A modification promotes tumor progression by regulating the activation state of various signaling pathways. By modifying mRNA or interacting with ncRNA, m^6^A modification regulates the tumorigenesis process of abnormal proliferation, glycolysis, metastasis, invasion, EMT, and protective autophagy in the development of GC. This paper reviews the latest research progress on m^6^A methylation in GC, in which m^6^A-modified targets and their signal transduction factors were identified as potential biomarkers for GC diagnosis and prognosis or treatment targets, thereby providing ideas for future research on the regulation of m^6^A methylation on the tumorigenesis pathway and the development of GC-targeted therapeutic drugs. However, the regulatory pathways and targets of m^6^A modification in GC are insufficiently explored, warranting additional research.

## Author contributions

GL, QF, CL, YP, JG, SL, YH, and HZ: contributed to this article with the design. GL, QF, and YP: literature search. GL, QF, CL, and YH: drafting. GL, QF, CL, YP, YH, and HZ: revision. GL, QF, CL, YP, YH, and HZ: editing. YH, and HZ: final approval. All authors contributed to the article and approved the submitted version.
